# We miss the opportunity: Pretreament of osteoporosis in a German trauma center

**DOI:** 10.1371/journal.pone.0207122

**Published:** 2018-11-12

**Authors:** Valentin Rausch, Andreas Schwarzer, Johannes W. Dietrich, Miriam Kaisler, Dominik Seybold, Jan Vollert, Thomas A. Schildhauer, Christoph Maier

**Affiliations:** 1 Department of General and Trauma Surgery, BG University Hospital Bergmannsheil, Bochum, Germany; 2 Department of Pain Medicine, BG University Hospital Bergmannsheil, Bochum, Germany; 3 Department of Endocrinology and Diabetes, Medical Hospital I, BG University Hospital Bergmannsheil, Bochum, Germany; Harvard Medical School, UNITED STATES

## Abstract

Osteoporosis remains a major health concern due to high incidence of fragility fractures followed by higher mortality and morbidity. Implementation of guidelines for diagnosis and treatment of osteoporosis is critically discussed internationally. Aim of this study was to evaluate implementation of these guidelines regarding diagnosis and therapy of osteoporosis in a developed western country. We hypothesized that (a) prior diagnosis of osteoporosis in patients with low-energy fractures is higher than the estimated incidence and (b) diagnosis and therapy of osteoporosis in patients with prior low-energy fractures is higher than in patients without prior low-energy fractures. 399 patients >60 years suffering low-energy-fractures of their spine, femur, humerus or forearm between 03/2014 and 04/2015 were recruited in a German trauma center. All received a standardized interview. In 21% (84/399) of all patients, osteoporosis was diagnosed prior to current admission. 34% (136/399) suffered a prior risk-fracture after age of 50. Of these, only 54% (73/136) reported about following dual-energy X-ray absorptiometry (DXA) to test for decreased bone-marrow-density with positive results in 68% (50/73). 38% (19/50) of these patients with fragility fractures and prior osteoporosis diagnosis received anti-osteoporotic medication. 66% (263/399) of all patients had no prior risk-fracture and were tested for osteoporosis by DXA in 36% (95/263), leading to positive results in 34% (32/95). 44% (14/32) of these patients received anti-osteoporotic medication. Applying FRAX, 33% of all patients showed a calculated 10-year-risk >20% for suffering a major osteoporotic fracture. 61% (83/136) of patients with a prior fracture had a 10-year-risk >20% of which 47% (39/83) patients received no prior DXA. Although guidelines recommend diagnosis and treatment of patients with low-energy fractures, opportunity for early treatment following risk fractures seems rarely used. Expedient risk assessment is necessary to indicate further diagnostics and therapy of osteoporosis to ensure adequate and efficient treatment for osteoporotic fractures.

## Introduction

Osteoporosis is a common systemic disease, defined by decreased bone density and deterioration of bone structure leading to fragility of bones and a higher susceptibility for fragility fractures [1]. Data about its prevalence is limited, studies based on insurance data estimate that 39% of the female population over 50 years may be affected [2]. In males, prevalence is estimated to be on a significantly lower level with 9.7% of the population over 50 years [2]. Major hallmark of osteoporosis is the occurrence of fragility fractures, especially of the proximal femur, forearm and the spine, resulting in a higher mortality rate in those patients [3,4]. Subsequent costs of osteoporosis for the health care system are assumed to be high and estimated to reach as much as €5.4 billion in Germany, constituting over 3% of the total health care cost [2]. Objective of (early) diagnosis of osteoporosis is selecting and treating patients to reduce their risk for future fractures. It has been shown that drug-therapy that alters bone remodeling can significantly reduce fragility fractures by 20–60% [5–8]. Besides socioeconomic aspects, prevention of fragility fractures can avoid risks of reduced quality of life and increased mortality linked to osteoporosis [3,9,10].

Specific risk factors are linked to a positive diagnosis of osteoporosis, such as prior fractures after low-energy trauma, medication with steroids, proton-pump inhibitors or antidepressants, hip-fractures in parents, accompanying diseases (diabetes, epilepsy, chronic obstructive pulmonary disease, ankylosing spondylitis, chronic heart failure, Cushing’s syndrome, thyrotoxicosis), smoking or underweight. Several tools are available to calculate the risk of suffering a fragility fracture in osteoporotic patients based on their risk factors. The fracture risk-assessment tool (FRAX) is a well-established tool to calculate the 10-year-risk of suffering a major osteoporotic fracture based on the bone marrow density or the body mass index (BMI). However, measurement of the bone-marrow-density via Dual-energy X-ray absorptiometry (DXA) has the highest accuracy for the diagnosis of osteoporosis and is an efficient tool for the calculation of the individual risk and to indicate further treatment based on the deviation from the reference T-score [11]. Hence, guidelines recommend to perform DXA if certain risk factors are present in the patients’ history or to indicate treatment if the clinical and radiographic features strongly suggest a present osteoporosis [12].

Therefore, from a scientific perspective, patients admitted to the hospital due to low-energy-fractures pose a unique opportunity to evaluate for osteoporosis and recommend its further treatment, since known risk factors are recorded during hospital stays regularly. Especially in trauma-units, admitted patients over the age of 50 suffering from a fracture should be considered to be at a higher risk for osteoporosis. Not only should those patients be further evaluated if they suffered a low-energy fracture, anti-osteoporotic treatment might also be indicated if patients with vertebral-body-fractures and fractures of the proximal femur show typical radiologic or clinical signs of osteoporosis [12]. However, the known gap between patients that have become clinically apparent and those that receive adequate diagnostics and therapy has not yet been successfully closed and patients still do not receive adequate diagnosis and treatment for osteoporosis. Aim of this study was to investigate osteoporotic diagnosis and treatment among patients over 60 years admitted to a German trauma center after suffering a low-energy-fracture and to compare the diagnostics and treatment between those that suffered a prior risk fracture over the age of 50 and those that did not.

## Materials and methods

Data for this retrospective investigation was collected within the FROP-study, evaluating opioid-medication as a risk factor for fractures in the elderly population. After approval of the local ethics committee (Reg. Nr.: 4876–13; Clinical Trials Nr.: NCT02090972), all patients above 60 years of age who were admitted to the Department of General and Trauma Surgery of the University Hospital Bergmannsheil Bochum between 03/2014 and 02/2015 after suffering a low-energy fracture were included in this study. Exclusion criteria were: malignant disease in the last 12 months, admittance to a hospital or a nursing home up to 14 days prior to admittance to our department, signs of mental impairment and diseases such as dementia or Parkinson’s disease and prior fracture in the last 6 months. For the original study, a control group without a low-energy fracture was recruited, which was not included in this study.

Patients were interviewed with a standardized questionnaire, including medication, comorbidities and other risk factors for osteoporosis (hip fracture of the parents, prior fractures with age >50 years, smoking, abuse of alcohol) among other questions. Risk fractures for osteoporosis were defined as all fractures except those of the ankle, hands, feet and the skull, since those fractures do not seem to be associated with a higher risk of osteoporosis [13]. Information of the interviews were compared with patients’ files of admittance or information from their general practitioner. With this information, the 10-year risk for suffering a major osteoporotic fracture was calculated based on the FRAX tool and using the BMI [14]. In order to estimate the statistical relation between patients with and without risk fractures, significance was calculated with use of the Chi-squared test (or Fisher’s exact test, if appropriate) and the Mann-Whitney-U test for count data and continuous variables, respectively. For the statistical relation between FRAX measures of different groups, significance was calculated with use of the Kruskal-Wallis-test. A p-value < 0.05 was considered significant, a p-value < 0.001 was considered highly significant. For statistical analysis and visualization, Microsoft Excel (Microsoft Corp., Redmond, WA, USA) and GraphPad Prism 7 (GraphPad Software, La Jolla, CA, USA) was used.

All procedures performed in studies involving human participants were in accordance with the ethical standards of the institutional and/or national research committee and with the 1964 Helsinki declaration and its later amendments or comparable ethical standards. All patients provided written consent to have their medical records used for this study after the aims and methods as well as the anticipated benefits and potential risks of the original study were explained to them.

## Results

In total, 399 (female: 296, 74%) patients admitted to the hospital were included, the median age was 75 years (Table 1).

**Table 1:** Demographic data, fractured region on admission, type of treatment received.

**Total**	399	
**Age**	median: 75; range: 60–101; SD: 9.33	
**Sex**	female: 296 (74.2%); male: 103 (28.8%)	
**Fracture on admission**	head	4
	trunk	58
	upper extremity	157
	lower extremity	180
**Conservative Treatment**	49 (12.3%)	
**Operative treatment**	350 (87.7%)	

21% (84) of these patients were aware of having a history of osteoporosis. 93% (78) of these were female. For diagnosis of osteoporosis, a prior DXA had been performed in 43% (168) of all patients. Of all DXA tested patients, 49% (82) reported about a pathologic result, indicating osteoporosis. In two patients, osteoporosis was diagnosed without DXA.

**Fig 1:**
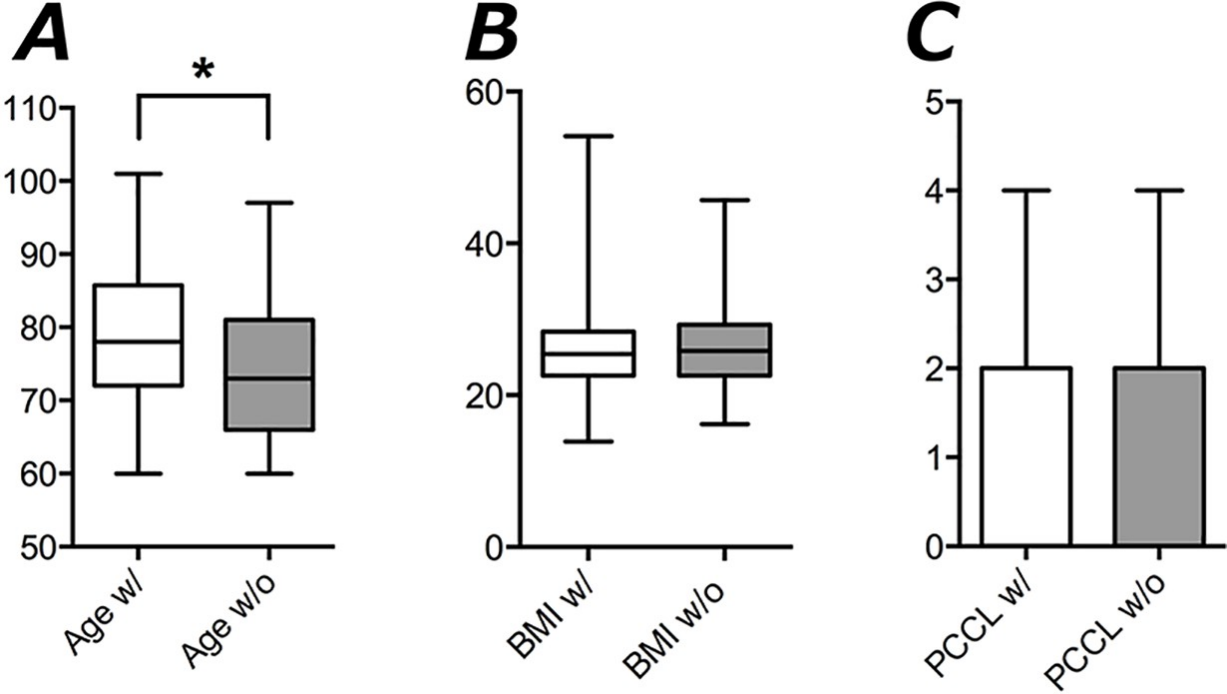
Boxplots (Mean, 25th to 75th percentiles, Min to max) of clinical data in patients with and without prior risk fractures: (* p < 0.05). (A) Age (B) Body mass index (BMI) (C) Patient Clinical Complexity Level (PCCL).

34% (136) of all patients had suffered a prior risk fracture after the age of 50 years. A majority of these patients (84%, 114) was female (Table 2), and patients with prior fracture were significantly younger compared to those without prior fractures (68 ± 5 vs. 83 ± 5 years, Fig 1). The mean in-patient stay and rate of complications as indicated by the Patient Clinical Complexity Level (PCCL) was not significantly different in patients with or without prior indicator fracture, respectively (Table 2). Patients without a prior risk fracture reported significantly higher mobility, less impairment of walking and sports and needed less help in their household. Also, patients without prior fractures used significant less opioids and tranquilizers (Table 2).

**Table 2:** Differences in demographics, comorbidities, mobility, diagnosis and treatment of osteoporosis in patients with and without prior risk fracture.

	prior fracture	no prior fracture	p-value
**total**	136	263	
**female**	83.8% (114)	69.2% (182)	*0.002
**mean stay in hospital (days)**	11.7±7.7	12.1±7.+3	0.553
**alcohol abuse**	0.0% (0)	2.7% (7)	0.055
**smoking**	38.2% (52)	41.1% (108)	0.585
			
**medication**			
**≥ tranquilizer**	16.9% (23)	8,4% (22)	*0.011
**≥ opioid**	20.6% (28)	9.5% (25)	*0.002
**≥ corticoid**	15.4% (21)	13.3% (35)	0.561
**≥ PPI**	50% (60)	37.3% (98)	*0.014
**≥ neuroleptics**	11.8% (16)	9.5 (25)	0.481
**≥ anti-diabetic drugs**	14.7% (20)	15.2% (40)	0.894
			
**use of walking aid**	44.9% (61)	26.6% (70)	*<0.0001
			
**mobility**			
**high mobility**	29.4% (40)	51.3% (135)	*<0.0001
**small impairment**	35.3% (48)	27.8% (73)	
**major impairment**	35.3% (48)	20.9% (55)	
**help in household**			
**no help needed**	35.3% (48)	61.6% (162	*<0.0001
**only housework**	36.8% (50)	23.2% (61)	
**help with personal hygiene**	27.9% (38)	15.2% (40)	
			
**sports (cycling, swimming, running)**			
**no impairment**	19.9% (27)	37.3% (98)	*0.0001
**possible with impairment**	27.9% (38)	25.9% (68)	
**not possible**	52.2% (71)	36.9% (97)	
**physical therapy and gymnastics**			
**no impairment**	38.2% (52)	55.9% (147)	*0.0001
**possible with impairment**	32.4% (44)	27.4% (72)	
**not possible**	29.4% (40)	16.7% (44)	
			
**DXA**	53.7% (73)	36.1% (95)	*<0.0001
**Pre-diagnosed osteoporosis**	36.8% (50)	12.9% (34)	*<0.0001
**Supplementation with Cholecalciferol (Vit. D)**	13.2% (18)	8.4% (18)	0.125
**Supplementation with Calcium**	5.1% (7)	4.9% (13)	0.929
**Therapy with Bisphosphonates**	13.2% (18)	3.4% (9)	*0.0002
**other anti-osteoporotic drugs**	0.7% (1)	0.8% (2)	0.978
			
**Mean hospital stay in days ± SD**	11.7 ± 7.7	12.1 ± 7.3	0.553
**Patient Clinical Complexity Level (PCCL) ± SD**	1.1 ± 1.43	0.97 ± 1.38	0.365

* p < 0.05

Of the 136 patients with a prior risk fracture for osteoporosis, only 54% (73) had been investigated for osteoporosis with DXA, resulting in a positive osteoporosis-diagnosis in 68% (50). The remainder of 263 patients that did not report about a prior risk fracture had received a DXA in 32% (95) with a positive rest-result in 11% (32) (Table 2).

**Fig 2:**
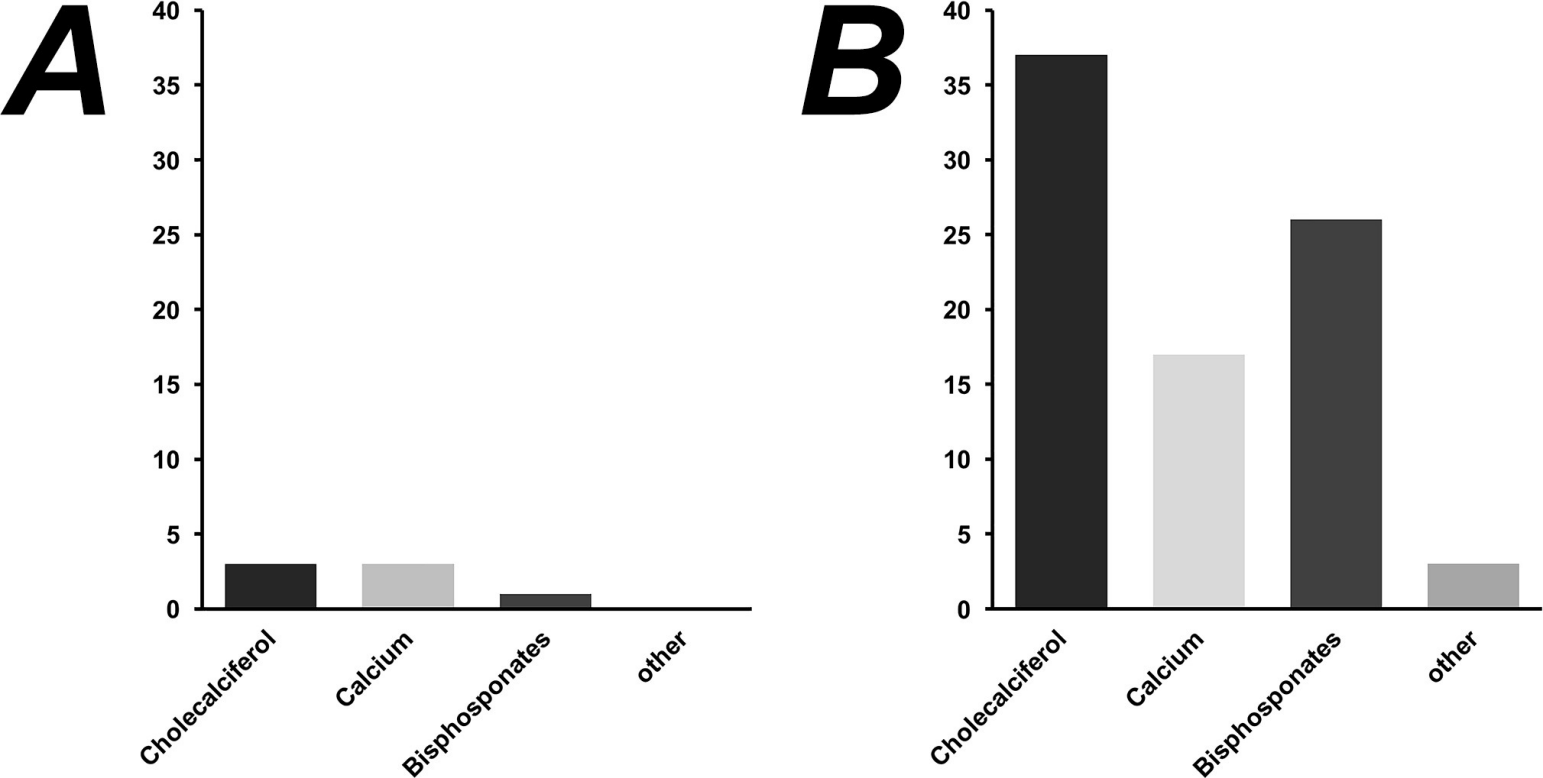
**Overall prescribed anti-osteoporotic medication in** (A) male (B) female patients.

Medication for osteoporosis was prescribed in only 15% (60) of all 399 patients (Fig 2). All patients on medication received supplementation of Cholecalciferol (Vitamin D) or Calcium. Additional antiresorptive medication was prescribed in 7% (28) of all patients. Of these, bisphosphonates were prescribed in 27 and strontium ranelate in one case. In two cases, patients received estrogen derivatives for therapy of osteoporosis.

Only a minority of patients diagnosed with osteoporosis received treatment: 40% (33) of patients that had been prior diagnosed with decreased bone density via DXA were treated with any medication for osteoporosis, whereas the remaining 60% (49) did not receive any anti-osteoporotic drug-therapy, including Calcium or Vitamin D3. Patients with a prior fracture received significantly (p = 0.005) more often medication for osteoporosis with 22% (30), compared to 11% (30) in the group without a prior fracture. Of note, 10% (9) of the 86 patients, which had been tested for osteoporosis with use of DXA and showed no sign of a decreased BMD, reported about receiving medication for osteoporosis anyway.

**Fig 3:**
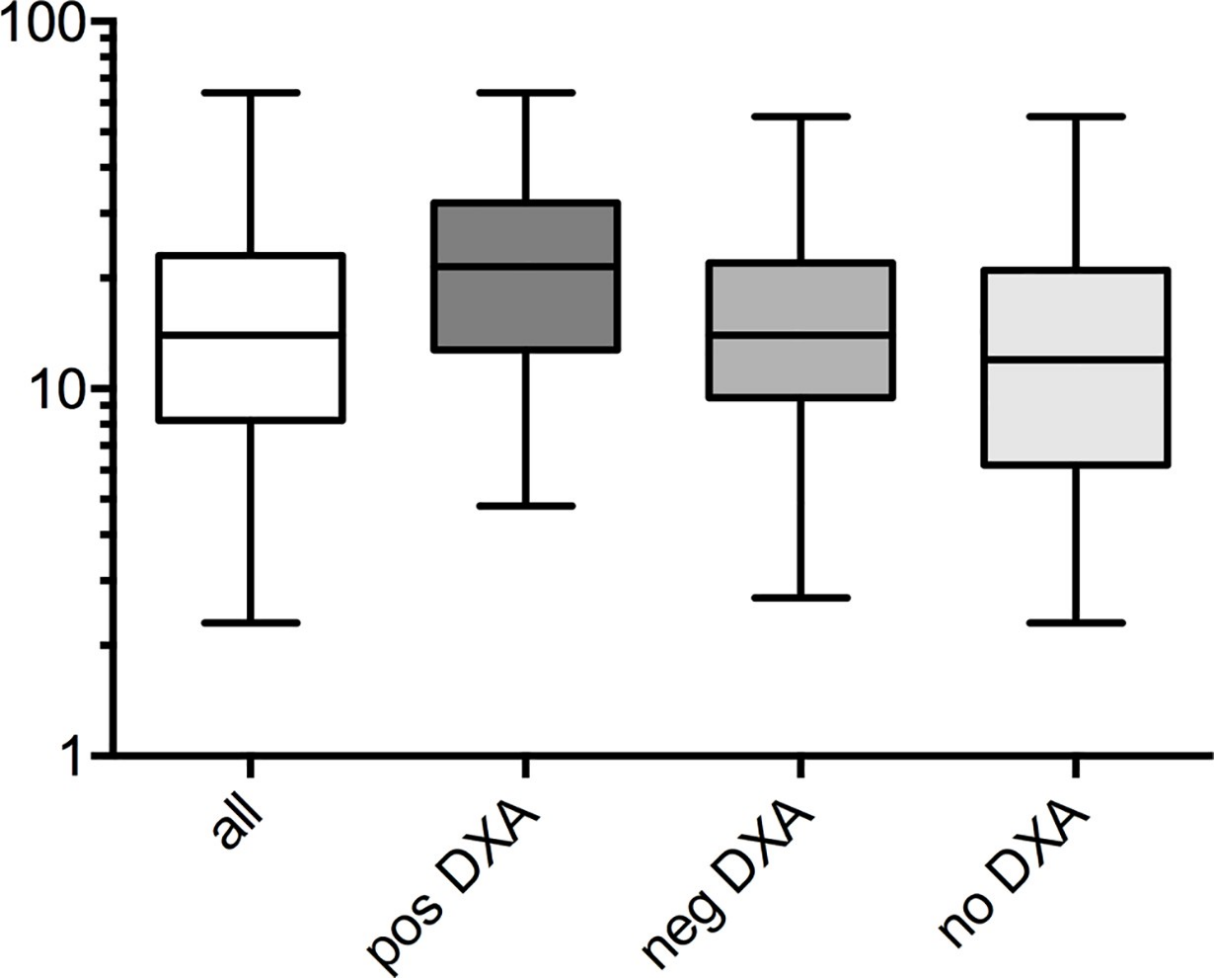
10-year probability (mean, min-to-max and standard deviation) based on the FRAX-tool in all patients, with positive and negative result in DXA and no BMD measurement using DXA.

Calculating the 10-year-risk for a major osteoporotic fracture according the FRAX-tool showed a mean risk of 16.90% in all patients. However, 32.8% (131) of all patients had a calculated 10-year-risk >20% for an osteoporotic fracture. Of patients with a prior risk fracture for osteoporosis, 61% (83) showed a risk >20%. Surprisingly, 47% (39) of these patients did not receive recommended measurement of BMD using DXA. Test for distribution of age and sex in patients depending on the calculated risk for major osteoporotic fractures revealed a significant difference between male and female patients (p<0.001) with a relative risk of 1.69 for the male population to have a calculated 10-year-risk for major osteoporotic fractures >10%. Also, a highly significant difference could be shown between patients below age 75 or 75 and above (p<0.001) with a relative risk of 2.05 for the younger population to have a calculated 10-year-risk for major osteoporotic fractures >10%. Calculating the 10-year-risk for major osteoporotic fractures revealed differences between patients depending if their BMD was measured or not. The calculated risk for a major osteoporotic fracture was significantly different between patients with a BMD indicating positive (23.37%, SD: 12.52) and negative (16.94%, SD: 10.83) diagnosis for osteoporosis or those that had not been tested for osteoporosis using BMD (14.60%, SD 10,26) (Fig 3).

**Fig 4:**
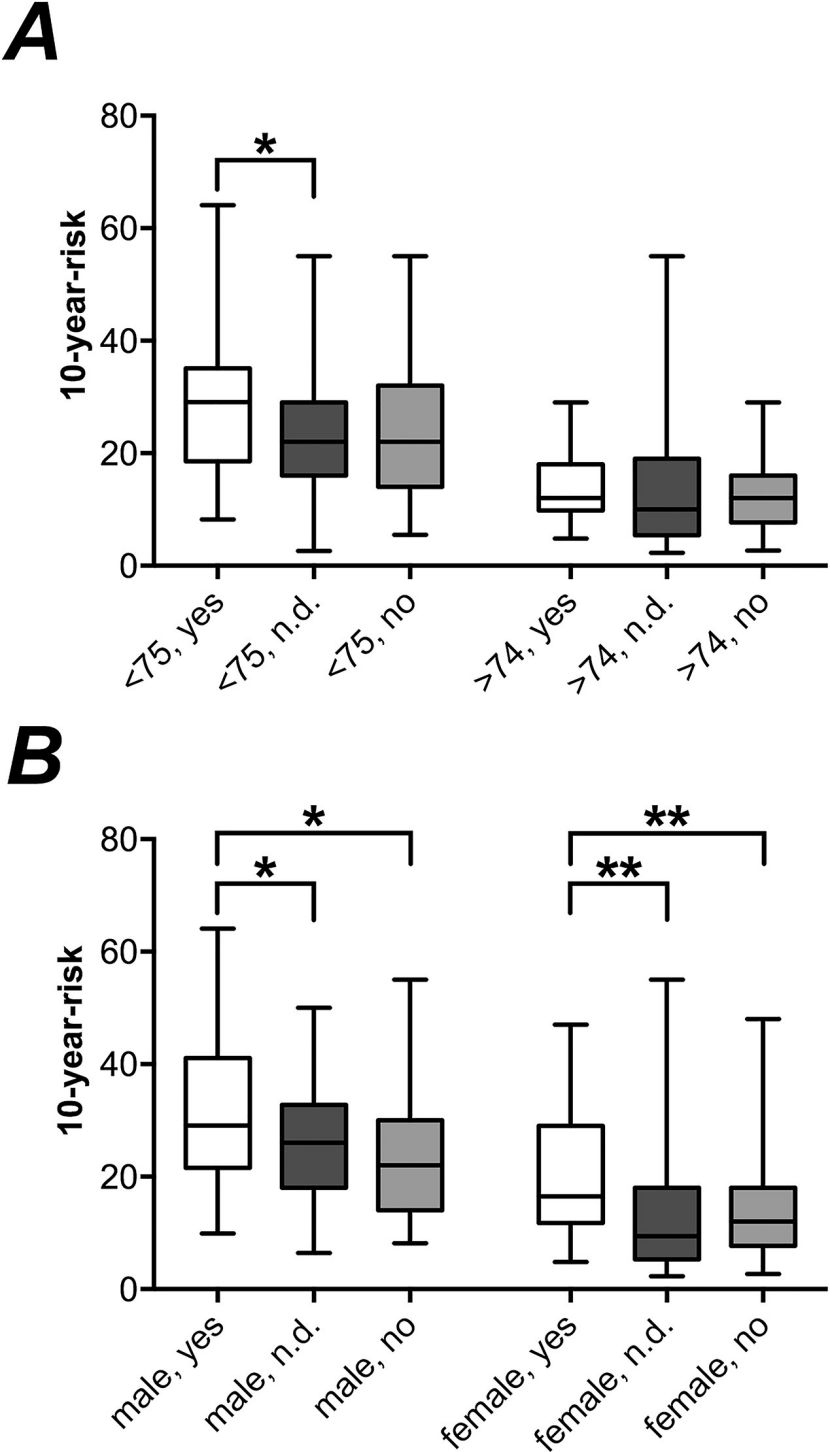
**10-year probability (mean, min-to-max and standard deviation) based on the FRAX-tool in all patients, with positive (yes) and negative (no) result in DXA and no BMD measurement using DXA (n.d.) divided by** (A) sex and (B) age (below 75 and above 74 years).

In female patients, differences in the calculated 10-year-risk for major osteoporotic fractures depending on whether the BMD was measured or not only revealed highly significant differences between positive (19.34%, SD: 10.1) and negative (13.20%, SD: 8.2) diagnosis (p<0.001) and between a positive diagnosis and those patients that had not been tested using BMD (12.01%, SD:8.4; p<0.001). In male patients, significant differences could be found between patients with a positive diagnosis (31.14%, SD: 2.5) and those patients that had not been tested using BMD (25.6%, SD: 1.5; p>0.05) and between patients tested positively and those that had not been tested using BMD (23.6%, SD: 2.1; p<0.05) (Fig 4).

When divided in patients below the age of 75 and those of 75 and above, significant differences in the calculated 10-year-risk for major osteoporotic fractures depending on whether the BMD was measured or not could be found between patients that did not receive BMD-measurement (22.88%, SD: 10.2) or had a positive result (28.51%, SD:12.1) (p = 0.021) in the younger age group (Fig 4). In patients with the age of 75 and above, significant differences could be found between patients that did not receive BMD-measurement (9.21%, SD: 5.6) and both, patients with a positive result (13.96%, SD:3.2) (p<0.001) and a negative result (12.30%, SD:6.1) (p = 0.002) (Fig 4).

## Discussion

In this retrospective investigation, we analyzed 399 patients admitted to our hospital after a low-energy fracture for their prior osteoporosis risk factors, diagnosis and treatment.

Of these patients, 36% (136) reported a prior risk fracture for osteoporosis after the age of 50. The results are consistent with that of a meta-analysis by Kanis et al., which showed the probability of a fracture history to rise almost linearly with age, and a risk of 30–45% in patients of between the age of 50–90 [15]. In younger patients, prevalence for prior fracture history is higher in males, whereas older women are more likely to report about prior fractures than older men. This is most likely due to the higher incidence of osteoporotic fractures in older women. Also, patients that suffered from a fracture show a significantly higher risk to suffer a subsequent fracture [15]. Male patients hospitalized with a fragility fracture showed a higher relative 10-year-risk to suffer a major osteoporotic fracture than women. Differentiated by age, younger patients (<75 years) had a higher relative 10-year-risk to suffer a major osteoporotic fracture than patients above 74 years.

However, surprisingly few patients were aware of an osteoporosis-diagnosis prior to admittance, which was also the case when a prior risk fracture was reported. In Germany, prevalence for osteoporosis in individuals of age 50 and above is estimated to be around 26% overall and 39% in women [2], while the rate of osteoporosis in our patients was only 21% overall and 26% in women, respectively. Particularly in elderly patients (of age 60 and above), hospitalized due to low-energy fractures, one would assume osteoporosis to occur much more frequently. One reason for this low prevalence in our study might be a diagnostic gap due to the low rate of patients tested for osteoporosis with DXA, which was underwent in only 47% of all patients and 36% in patients with a prior indicator fracture. For the latter, missing diagnostics seem particularly surprising, since they were admitted regularly to a hospital due to said fracture. Furthermore, newest guidelines would suggest treatment without measurement of BMD if patients show osteoporotic-typical fractures. Yet, of all DXA-tested patients, only 49% reported a pathological result indicating osteoporosis and only 40% of patients with a positive DXA-test received drug-therapy for osteoporosis.

According to the current guidelines, routine-measurement of BMD should only be indicated if the overall risk for a vertebral body-fracture or a hip fracture is thought to be >20%. One well-established method for the calculation of the 10-year-risk is the FRAX tool, based on either the BMD or the BMI. When calculating the 10-year-risk for our patients suffering a major osteoporotic fracture using the FRAX-tool, only patients that were tested for osteoporosis and had a positive result showed a calculated risk >20%. Patients that had not been tested as well as patients that were tested negatively for osteoporosis showed a calculated risk of 14.60% and 16.94%, respectively. This also applies when corrected for age or sex. Therefore, prior indication for osteoporotic diagnostics seems in accordance with the current guidelines. In light of the frequency of fragility fractures in our population however, earlier diagnosis and treatment in those patients should be considered carefully. Our data suggest that especially younger patients (<75 years) and male patients hospitalized due to a fragility fracture should be thoroughly investigated for osteoporosis and treated accordingly.

Taken together, although diagnostics for osteoporotic in patients suffering a risk-fracture was indicated according to the risk proposed in the current guidelines, the measures resulted in a lack of treatment in this disease. Patients need to be thoroughly evaluated for therapeutic options once diagnosed. Underdiagnosis and undertreatment of osteoporosis are well-known phenomena: Although the socioeconomic burden is high and adverse effects in affected patients are significant, a large proportion is assumed to neither be diagnosed correctly nor receiving adequate treatment [16–18]. Gardner [18] retrospectively investigated 75 patients between 1997 and 2000 admitted with low-energy fractures and found only 11% were treated with anti-osteoporotic medication on discharge, thus concluding patients were undertreated for osteoporosis. Panneman [16] analyzed 1654 patients with osteoporotic fractures for use of anti-osteoporotic drugs: Only 14.9% of these patients had been prescribed anti-osteoporotic drugs within 1 year after discharge from the hospital, 6.8% of these patients received bisphosphonates. This data is consistent with our observations. Despite advances in the guidelines in Germany, numbers of prescription in our study are the same as found by Panneman in 2003 [16].

Reasons for this observation might be the costs of measuring BMD with use of DXA which are not necessarily reimbursed by the statutory health insurance in Germany. Since a decreased BMD is usually recommended in advance of anti-osteoporotic therapy, patients missing DXA do not receive therapy in the majority of cases.

Aim of the orthopedic surgeon should however be the selection of patients in whom treatment promises future fracture prevention, since osteoporosis treatment has shown to be efficient in terms of reducing mortality and improving quality of life. This especially applies for patients suffering a low-energy vertebral or hip fracture, since osteoporosis has already caused a fragility fracture in these patients. Various options for a risk assessment in these patients are available, such as FRAX [14], the DVO-risk model 2006 [19] or the Q-fracture score [20], based solely on clinical aspects.

Taken together, our study could show, that further improvements of risk assessment measures to detect patients with osteoporosis are still needed in the clinical routine. Implementation of existing guidelines in all disciplines participating in the treatment of patients with osteoporosis are of utmost importance to indicate diagnosis and following treatment. Therefore, during hospital stay of patients with fractures suspicious for osteoporosis, we recommend further diagnostics after performing a risk-assessment, acknowledging the abovementioned risk factors. Also, following the newest guidelines, we recommend treatment without DXA in patients where clinical signs of radiological changes are typical for osteoporosis and contraindications for drug-therapy are missing. These interdisciplinary approaches are vital for more promising future results.
